# Automated Longitudinal Quantification of Retinal and Choroidal Vascular Changes After Phacoemulsification

**DOI:** 10.3390/tomography12030042

**Published:** 2026-03-19

**Authors:** Seung Hoon Lee, Phil Kyu Lee, Se Eun Park, Ho Ra, Jiwon Baek

**Affiliations:** 1 Department of Ophthalmology, Bucheon St. Mary’s Hospital, College of Medicine, The Catholic University of Korea, #327 Sosa-ro, Wonmi-gu, Bucheon 14647, Gyeonggi-do, Republic of Korea; 1124mynamelsh@gmail.com (S.H.L.); feeelfal@gmail.com (P.K.L.); pse9633@naver.com (S.E.P.); raho@catholic.ac.kr (H.R.); 2 Department of Ophthalmology, College of Medicine, The Catholic University of Korea, 222 Banpo-daero, Seocho-gu, Seoul 06591, Republic of Korea

**Keywords:** phacoemulsification, cataract, vessel density, retina, choroid, clinical factors, association

## Abstract

Background/Objectives: To comprehensively evaluate longitudinal retinal and choroidal vascular changes after phacoemulsification using automated optical coherence tomography angiography (OCTA) analysis and to investigate clinical factors influencing these changes. Methods: This retrospective study included 26 subjects (31 eyes) who underwent uncomplicated phacoemulsification. OCTA was performed at baseline and at 1 day, 1 week, 1 month, and 2 months postoperatively. Automated quantitative analysis was applied to assess vessel density- and structure-related parameters in the superficial capillary plexus (SCP), deep capillary plexus (DCP), choriocapillaris, and Haller layer. Longitudinal changes were analyzed using repeated-measures analysis of variance, with time × clinical factor interactions evaluated for diabetes mellitus, anesthesia method, and sex. Inter-layer associations were assessed using Spearman correlation analysis. Results: Significant longitudinal changes were observed in retinal layers. In the SCP, vessel density increased from 42.59 ± 1.46 at baseline to 44.10 ± 1.44 at 2 months (*p* = 0.002), accompanied by increases in vessel length and node counts (all *p* < 0.001). In the DCP, vessel density increased from 34.66 ± 5.98 to 38.65 ± 4.83 (*p* < 0.001). In contrast, choriocapillaris-related parameters showed no significant overall time effect. In the Haller layer, mean vessel diameter decreased significantly over time (*p* < 0.001), while density-related metrics remained unchanged. ΔVAD demonstrated positive correlations between adjacent layers (SCP–DCP and DCP–choriocapillaris) and a negative correlation between choriocapillaris and Haller layers. Diabetes mellitus showed no significant longitudinal effect, whereas retrobulbar anesthesia and sex significantly modified selected choroidal trajectories. Conclusions: Automated and integrated OCTA analysis revealed layer-dependent retinal and choroidal vascular responses after phacoemulsification, with coordinated changes confined mainly to anatomically adjacent layers and selective modulation by clinical factors.

## Simple Summary

We investigated how small blood vessels in the retina and choroid change after uncomplicated cataract surgery using advanced imaging. Retinal vessel density and structural complexity increased over two months, while deeper choroidal vessel caliber showed subtle changes. The results suggest that cataract surgery may be associated with microvascular alterations after accounting for image quality, providing insight into postoperative vascular dynamics and informing future research on ocular vascular health.

## 1. Introduction

Cataract is one of the leading causes of visual impairment worldwide, and phacoemulsification with intraocular lens implantation is the most commonly performed surgical procedure in ophthalmology [1]. Beyond visual rehabilitation, cataract surgery has been reported to induce postoperative alterations in ocular blood flow, potentially related to functional hyperemia, changes in light transmission, and postoperative inflammatory responses [2]. Previous studies have described postoperative changes in retinal and choroidal circulation; however, most investigations relied on optical coherence tomography (OCT)–based measurements of retinal or choroidal thickness or on choroidal vascular index–based assessments.

Optical coherence tomography angiography (OCTA) enables noninvasive, depth-resolved visualization of retinal and choroidal vasculature without the need for dye injection [3]. OCTA allows quantitative evaluation of vascular parameters, including vessel density and structural metrics, across distinct vascular layers [4,5]. Despite these advantages, OCTA-based studies evaluating longitudinal vascular changes after cataract surgery remain limited, and most have focused on either retinal or choroidal compartments in isolation [3,6].

In our previous work, we investigated clinical factors affecting postoperative changes in choroidal thickness and choroidal vascularity index using enhanced-depth OCT [7]. Building on these findings, the present study aimed to perform a comprehensive, automated, and longitudinal OCTA-based analysis of retinal and choroidal vascular changes following uncomplicated phacoemulsification. In particular, we sought to characterize layer-specific vascular changes over time, assess correlations between retinal and choroidal vascular layers, and evaluate the influence of clinical factors on longitudinal OCTA parameter trajectories.

## 2. Materials and Methods

We retrospectively reviewed the medical records of patients who underwent phacoemulsification and posterior chamber intraocular lens implantation for simple cataract without complications at Bucheon St. Mary’s Hospital, The Catholic University of Korea, from March 2022 to October 2022. This study was conducted with approval of the Institutional Review Board (IRB) of Bucheon St. Mary’s Hospital (IRB no. HC23RASI0003) and was exempt from requiring patient research consent. All procedures were completed in accordance with the Declaration of Helsinki. Patients with simple cataracts were included, and exclusion criteria were: high myopia or axial length extremes (axial length > 26.0 mm or spherical equivalent < −6.0 D); a history of glaucoma or ocular hypertension; any macular pathology; and a history of retinal surgery, laser treatment, or any intraoperative complications. Individuals with poorly controlled systemic vascular diseases or advanced diabetic retinopathy were excluded. Eyes that completed all scheduled follow-up visits and met predefined OCTA image quality criteria or segmentation failure at all time points were included in the final analysis.

Cataract surgery was performed by a skilled operator with over 10 years of surgical experience. All intraocular lenses used were Tecnis 1-Piece aspheric acrylic posterior chamber intraocular lenses (IOL; ZCB00^®^; Johnson & Johnson, New Brunswick, NJ, USA). All operations were completed within 10 to 20 min without any surgical complications. Anesthesia was administered as either topical or retrobulbar anesthesia according to patient preference and cooperation. Topical anesthesia was performed by instilling 2–5 drops of proparacaine hydrochloride 0.5% (Alcaine^®^; Novartis, Basel, Switzerland) into the surgical eye immediately before surgery. For retrobulbar anesthesia, lidocaine hydrochloride 2% (Lidocaine^®^; Huons, Seongnam-si, Gyeonggi-do, Republic of Korea) and bupivacaine hydrochloride 0.5% (Pucaine^®^; Reyon, Seoul, Republic of Korea) were mixed in a 7:3 ratio, and 1.5–2 mL of the mixture was injected into the retrobulbar space, followed by ocular compression for 10–15 min using a reducer. A 2.4 mm clear corneal incision was created using a Clear cut^®^ blade (Alcon, Geneva, Switzerland). Hyaluronic acid 1.5% (Hyalu^®^; Hanmi, Seoul, Republic of Korea) was injected into the anterior chamber as a viscoelastic material, and a continuous circular capsulorhexis was performed. Hydrodissection and hydrodelineation were carried out using balanced salt solution (BSS^®^; Alcon, Geneva, Switzerland). Lens emulsification was performed using the Centurion^®^ system (Alcon, Geneva, Switzerland), followed by irrigation and aspiration. The intraocular lens was then loaded into a cartridge and injector and implanted into the capsular bag. Postoperatively, 0.5% moxifloxacin hydrochloride (Vigamox^®^; Novartis, Basel, Switzerland) and prednisolone acetate 1% (Predforte^®^; Allergan, Dublin, Ireland) were instilled into the operated eye four times daily for 2 months.

**Figure 1:**
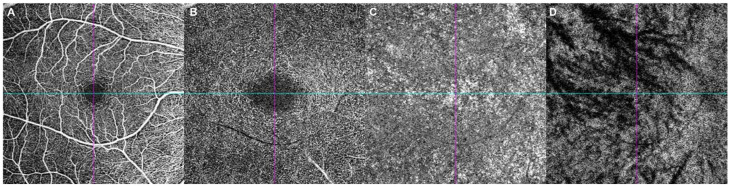
Representative en face OCT angiography images of retinal and choroidal vascular layers. Representative 6 × 6 mm en face OCT angiography images obtained using the Cirrus HD-OCT 6000 AngioPlex system are shown. En face OCT angiography images of the (**A**) superficial capillary plexus (SCP), (**B**) deep capillary plexus (DCP), and (**C**) choriocapillaris (CC), as well as an en face structural OCT image of the (**D**) Haller layer, are presented. The Haller layer was defined as the slab obtained at the level corresponding to half of the total choroidal thickness. All layers were extracted and processed using an automated analysis pipeline. Cyan and purple lines indicate horizontal and vertical lines crossing fovea, respectively.

All patients underwent 6 × 6 mm^2^ macular OCTA imaging (Cirrus HD-OCT 6000 Angioplex; Carl Zeiss Meditec, Jena, Germany) preoperatively and at 1 day, 1 week, 1 month, and 2 months after surgery. En face angiographic image slabs of the superficial capillary plexus (SCP), deep capillary plexus (DCP), and choriocapillaris, as well as en face structural slabs of the Haller layer (defined as the slab obtained at the level corresponding to half of the total choroidal thickness), were extracted using the Cirrus Review Software (ver. 11.5.2; Carl Zeiss Meditec) and saved as 412 × 412 pixel “.JPEG” files (Figure 1). JPEG compression settings were vendor-determined. All OCTA scans were acquired using the device’s built-in motion correction. Segmentation boundaries were visually reviewed, and scans with gross segmentation errors were excluded rather than manually corrected. Images with substantial artifacts or segmentation failures were excluded. Signal strength was recorded at each time point, and all baseline scans had ≥8.

All quantitative analyses were conducted using the OCTA Vascular Analyzer (OCTAVA) toolbox implemented in MATLAB R2024a (The MathWorks, Inc., Natick, MA, USA) [8]. OCTAVA was selected for its proven ability to provide standardized and reproducible extractions of multi-layer vascular metrics through an automated framework [9,10]. For the SCP, DCP and the Haller layer, images were processed using median filtering (filter size = 4), followed by adaptive local thresholding with a kernel size of 160, and skeletonization with a twig size of 4 to preserve continuous vessel structures and extract vessel centerlines. For the choriocapillaris, images were processed using median filtering (filter size = 3) and adaptive local thresholding with a smaller kernel size of 40, followed by skeletonization with a twig size of 3, optimized for dense microvascular patterns. OCTAVA automatically extracted multiple vascular parameters, including node count, vessel area density (VAD), total vessel length, vessel length density (VLD), mean vessel diameter, and mean tortuosity (Supplementary Table S1). VAD represents the proportion of vascular area within the scan region, VLD reflects skeletonized vessel length density, Nodes indicate branching complexity, and Mean Diameter represents average vessel caliber estimated using a local thickness algorithm. Vessel diameters were estimated using a local thickness algorithm applied to binarized images, while length-related metrics were derived from skeletonized images. Based on our previous assessments, en face structural slabs were selected for the Haller layer to ensure more accurate morphological analysis of large vessels against signal attenuation, while skeletonization-based metrics for the choriocapillaris were employed to provide a standardized quantitative representation of microvascular structural integrity [9]. All analyses were performed using an automated batch-processing workflow to minimize observer-dependent variability.

Clinical factors potentially affecting vessel density, including the method of anesthesia and the presence of diabetes mellitus or hypertension, were retrospectively collected through medical record review.

Statistical analysis was performed using MATLAB 2024a and Python 3.9. Longitudinal changes in vascular parameters across postoperative time points (baseline, 1 day, 1 week, 1 month, and 2 months) were primarily analyzed using linear mixed-effects models. Time was modeled as a fixed effect, and time × clinical factor interaction terms were included to assess the influence of diabetes mellitus, anesthesia method, sex, and signal strength (as a time-varying covariate) on longitudinal trajectories. To account for potential inter-eye correlation, the subject was included as a random intercept in all models. Adjusted β coefficients with 95% confidence intervals were reported. Repeated-measures analysis of variance (RM-ANOVA) was performed only for descriptive comparison. False discovery rate (FDR) correction was applied within each family of related hypotheses. Statistical families were defined within each clinical factor across all layer–parameter combinations. For correlation analyses, changes in vascular parameters at each postoperative time point relative to baseline were calculated, and Spearman correlation coefficients were used to evaluate inter-layer associations between retinal and choroidal vascular changes. When appropriate, paired *t*-tests were applied for descriptive comparisons between baseline and individual postoperative measurements. *p*-values were adjusted using the Benjamini–Hochberg FDR procedure. A *p*-value < 0.05 was considered statistically significant.

## 3. Results

### 3.1. Baseline Demographics and Clinical Characteristics

A total of 26 (31 eyes) were included, of whom 5 subjects contributed both eyes. The mean age was 72.5 ± 8.9 years. Females accounted for 21 eyes (67.7%) and males for 10 eyes (32.3%). Diabetes mellitus was present in 12 eyes (38.7%), and retrobulbar anesthesia was performed in 4 eyes (12.9%). Right eyes comprised 14 eyes (45.2%) and left eyes 17 eyes (54.8%) (Table 1). Signal strength increased significantly after surgery (baseline: 9.13 ± 0.92; 1 week: 9.84 ± 0.52; 1 month: 9.90 ± 0.30; 2 months: 9.90 ± 0.30; *p* < 0.001).

### 3.2. Layer-Specific OCTA Parameter Changes over Time

Layer-wise longitudinal changes were primarily evaluated using linear mixed-effects models (Table 2). RM-ANOVA results are provided for descriptive consistency. In the SCP, Mean Diameter decreased from 31.677 ± 0.832 at baseline to 30.800 ± 0.913 at 2 months (*p* < 0.001), while VAD increased from 42.590 ± 1.462 to 44.097 ± 1.443 (*p* = 0.002), and VLD (%) increased from 18.046 ± 1.018 to 19.411 ± 1.232 (*p* < 0.001). Mean Tortuosity showed a small but significant reduction over time (*p* = 0.008). Structural complexity measures increased significantly: Nodes rose from 2146.387 ± 177.517 to 2419.800 ± 216.440 (*p* < 0.0001), and Total Length increased from 369.056 ± 20.796 to 396.936 ± 25.187 (*p* = 0.0001).

In the DCP, robust postoperative increases were observed. VAD increased from 34.659 ± 5.984 at baseline to 38.645 ± 4.825 at 2 months (*p* < 0.001), and VLD (%) increased from 6.839 ± 1.329 to 7.682 ± 1.188 (*p* < 0.001). Nodes increased from 2308.290 ± 774.577 to 2711.400 ± 791.607 (*p* < 0.001), and Total Length increased from 341.989 ± 66.431 to 384.090 ± 59.353 (*p* < 0.0001).

In the choroid, none of the key parameters demonstrated a statistically significant overall time effect by RM-ANOVA (all ≥0.08) in the choriocapillaris layer. In the Haller layer, Mean Diameter decreased significantly from 97.161 ± 10.162 at baseline to 93.083 ± 7.790 at 2 months (*p* < 0.001).

### 3.3. Correlation Between Layer-Specific Changes

Spearman correlations were computed using layer-specific change values defined as Δ = (2 months − baseline). For ΔVAD, significant inter-layer associations were observed: a positive correlation between the DCP and SCP (r = 0.504, *p* = 0.010), a positive correlation between the DCP and choriocapillaris (r = 0.532, *p* = 0.006), and a negative correlation between the choriocapillaris and Haller layer (r = −0.647, *p* = 0.001) (Table 3 and Supplementary Table S2).

### 3.4. Differences in Change by Clinical Factors

Overall time–slope interaction analyses demonstrated no significant differences in longitudinal OCTA parameter changes according to diabetes mellitus status across the evaluated metrics (all *p* ≥ 0.05). In contrast, retrobulbar anesthesia was associated with a significantly different longitudinal trajectory of choriocapillaris VAD (β = −0.526, *p* = 0.015). Sex significantly modified longitudinal OCTA changes across multiple layers. In the choriocapillaris, significant time × sex interactions were observed for VAD (β = −0.366, *p* = 0.034) and Nodes (β = −38.76, *p* = 0.028). In the DCP, Mean Diameter showed a significant sex-related interaction (β = −0.224, *p* = 0.031), though the association was less pronounced compared to density-related parameters. In the Haller layer, sex was associated with differential longitudinal changes in structural parameters, including Nodes (β = 12.86, *p* = 0.009), Total Length (β = 2.66, *p* = 0.011), VAD (β = 0.382, *p* = 0.013), and VLD (%) (β = 0.130, *p* = 0.014) (Table 4 and Supplementary Table S3).

When signal strength was included as a time-varying covariate, the longitudinal change in parameters (VAD, VLD, Nodes, and Total Length) remained statistically significant (all *p* < 0.01). Sensitivity analyses excluding the early postoperative time point (1 day) confirmed that these longitudinal trends and their associations with clinical factors remained robust. Key mixed-effects model estimates (β with 95% CI) for representative retinal parameters are summarized in the Results section, with complete outputs provided in Supplementary Tables S3 and S4, and Supplementary Figure S1. These subgroup analyses should be considered secondary and exploratory.

## 4. Discussion

In this study, we performed an automated and longitudinal OCTA–based analysis of retinal and choroidal vascular changes following phacoemulsification and investigated clinical factors associated with these changes. The automated analysis approach minimizes inter-observer variability and improves measurement repeatability, enabling robust longitudinal comparisons. In addition, it allows comprehensive quantitative evaluation of multiple vascular parameters, including density-, length-, diameter-, and structure-related metrics, across retinal and choroidal vascular layers. By simultaneously assessing both retinal and choroidal vessels, this study provides a comprehensive characterization of postoperative vascular changes that have been insufficiently explored in previous OCTA-based cataract surgery studies.

OCTA provides quantitative information on retinal and choroidal vascular status, and several studies have reported OCTA-detected vascular changes after cataract surgery [11,12,13,14]. However, most previous studies were limited to a robust analysis of vessel density or thickness of the retinal layer. In contrast, the present study applied an automated analysis framework, quantified a broad range of detailed vessel metrics beyond vessel density, and comprehensively analyzed both retinal and choroidal vascular layers within the same cohort. In addition, inter-layer correlations and the influence of clinical factors on longitudinal vascular changes were systematically evaluated. This comprehensive approach distinguishes the present study from prior OCTA-based investigations of cataract surgery.

In the present study, significant longitudinal changes were observed in retinal vascular parameters, particularly in the SCP and DCP, with postoperative increases in vessel density– and structure-related metrics. These findings are consistent with previous OCTA studies reporting increased macular perfusion after phacoemulsification, with changes typically emerging from 1 week postoperatively [12,15,16,17,18]. While some studies have described plateauing or partial regression of retinal vascular changes by 3 months, our results demonstrated sustained increases in SCP and DCP parameters up to 2 months, which may reflect differences in follow-up duration and analytical approaches [13]. The underlying mechanisms for these postoperative OCTA changes remain uncertain; however, both functional hyperemia induced by increased retinal light stimulation after cataract removal and improved vessel detectability due to reduced media opacity represent plausible explanations for the observed findings [13].

In the choroidal layers, longitudinal analyses demonstrated no significant changes in choriocapillaris-related OCTA parameters, indicating relative stability of the choriocapillaris after phacoemulsification. In contrast, the Haller layer showed significant longitudinal changes confined to structural parameters, while density-related metrics remained unchanged. This pattern suggests that postoperative choroidal responses preferentially involve remodeling of large choroidal vessels rather than global changes in choroidal perfusion. Previous studies using choroidal vascular index have similarly reported postoperative shifts in the luminal–stromal balance without consistent or sustained changes in overall choroidal thickness or flow metrics [1,2,7,19,20,21]. In this context, the observed Haller layer changes may reflect subtle alterations in choroidal vascular composition rather than true perfusion loss. Possible mechanisms include postoperative modulation of choroidal vascular tone, altered light exposure following cataract removal, or redistribution between luminal and stromal components. The absence of concurrent choriocapillaris changes supports a localized, vessel-size–dependent response within the deeper choroidal circulation.

Correlation analyses revealed layer-specific and spatially constrained associations in postoperative vascular changes. ΔVAD showed positive correlations between adjacent layers (SCP–DCP and DCP–choriocapillaris), indicating coordinated density changes across contiguous retinal and choriocapillaris compartments [22,23]. In contrast, choriocapillaris ΔVAD exhibited a strong negative correlation with Haller layer ΔVAD, suggesting an oppositional response between superficial choroidal microvasculature and deeper choroidal vessels. Since prior studies have largely evaluated retinal or choroidal changes separately, information on correlations between these tissues remains limited. The present study suggests that although postoperative vascular alterations may occur globally, measurable correlations are predominantly confined to anatomically adjacent layers. While suggesting a linked response, these correlations should be interpreted cautiously as potential physiological autoregulation or blood flow redistribution rather than definitive causal remodeling.

Clinical factor analyses revealed selective influences on longitudinal OCTA parameter changes. Diabetes mellitus was not associated with significant differences in temporal trajectories across the evaluated retinal and choroidal metrics [24]. In contrast, retrobulbar anesthesia was associated with a significantly different longitudinal trajectory of choriocapillaris VAD, indicating a localized modulation of choriocapillaris-related vascular behavior by perioperative factors. Sex was a significant modifier of longitudinal vascular changes across multiple layers, including choriocapillaris- and DCP-related parameters, with more pronounced effects observed in the Haller layer. Sex-related effects likely reflect differences in choroidal vascular reactivity rather than disease-related microvascular damage. However, given the lack of formal inter- or intra-observer reproducibility analysis for Haller slab selection and the small number of eyes that received retrobulbar anesthesia, related findings should be interpreted cautiously and considered exploratory.

Several limitations of this study should be acknowledged. First, the sample size is relatively small, which may have limited the statistical power to detect subtle effects, and the follow-up period was limited to 2 months, representing short-term change only, which may change or plateau over time. Thus, our findings should be interpreted as exploratory and hypothesis-generating rather than confirmatory. Second, detailed information regarding diabetes type, disease duration, HbA1c levels, and severity of diabetic retinopathy was not available, which may have limited the ability to detect more subtle metabolic influences on postoperative vascular changes. Third, although most vascular parameters were derived using an automated workflow, delineation of the Haller layer required manual definition, which may introduce operator dependency. Because a formal intra- or inter-grader reproducibility analysis was not performed in the present dataset, Haller-layer–related findings should be interpreted cautiously and considered exploratory or hypothesis-generating. Fourth, subgroup analyses (e.g., anesthesia and sex) are exploratory due to small sample sizes. Potential confounding by indication cannot be ruled out, and future studies with larger cohorts are necessary to confirm these clinical associations. Additionally, while we used the vendor-defined JPEG export format for consistency across all images, future studies utilizing higher-resolution or lossless image formats may provide even more refined insights into pixel-level vascular changes. Despite these limitations, this study provides clinically meaningful insights into postoperative retinal and choroidal vascular dynamics.

## 5. Conclusions

In conclusion, we demonstrated significant longitudinal changes in retinal vascular parameters within the superficial and deep capillary plexuses, while the choriocapillaris remained largely stable using an automated and quantitative OCTA-based approach. In contrast, the Haller layer exhibited selective structural changes over time, suggesting a layer-specific response within the choroidal circulation. Notably, postoperative vascular trajectories were influenced by anesthesia and sex, indicating that clinical variables may play roles in shaping short-term vascular responses. In addition, simultaneous analysis of retinal and choroidal layers revealed layer-dependent and spatially constrained vascular responses after phacoemulsification.

## Figures and Tables

**Table 1:** Demographics and clinical characteristics.

Variable	Value
No. of eyes	31
Age, years (mean ± SD)	72.5 ± 8.9
Female, n (%)	21 (67.7%)
Male, n (%)	10 (32.3%)
Diabetes mellitus, n (%)	12 (38.7%)
Retrobulbar anesthesia, n (%)	4 (12.9%)
Right eye, n (%)	14 (45.2%)

Values are presented as mean ± standard deviation or number (%), as appropriate. DM = diabetes mellitus.

**Table 2:** Longitudinal Changes in Retinal and Choroidal Vascular Parameters After Phacoemulsification.

Layer	Parameter	Baseline	1 Day	1 Week	1 Month	2 Months	*p*-Value
SCP	Mean Diameter	31.677 ± 0.832	31.065 ± 1.459	30.806 ± 0.980	30.839 ± 1.003	30.800 ± 0.913	≤0.001
	VAD	42.590 ± 1.462	44.098 ± 2.599	43.764 ± 1.448	43.743 ± 1.572	44.097 ± 1.443	0.002
	VLD (%)	18.046 ± 1.018	19.088 ± 1.732	19.214 ± 1.277	19.118 ± 1.281	19.411 ± 1.232	≤0.001
	Mean Tortuosity	1.123 ± 0.005	1.122 ± 0.005	1.119 ± 0.003	1.119 ± 0.004	1.120 ± 0.003	0.008
	Nodes	2146.387 ± 177.517	2378.484 ± 310.517	2375.806 ± 219.624	2376.452 ± 229.588	2419.800 ± 216.440	≤0.001
	Total Length	369.056 ± 20.796	390.347 ± 35.413	392.913 ± 26.107	390.983 ± 26.196	396.936 ± 25.187	≤0.001
DCP	Mean Diameter	28.065 ± 1.548	28.000 ± 1.155	27.871 ± 1.204	27.935 ± 1.504	27.920 ± 1.352	0.458
	VAD	34.659 ± 5.984	37.307 ± 6.157	38.242 ± 5.912	37.922 ± 5.598	38.645 ± 4.825	≤0.001
	VLD (%)	6.839 ± 1.329	7.459 ± 1.441	7.658 ± 1.399	7.610 ± 1.322	7.682 ± 1.188	≤0.001
	Nodes	2308.290 ± 774.577	2559.677 ± 818.093	2653.548 ± 778.031	2613.903 ± 743.127	2711.400 ± 791.607	≤0.001
	Total Length	341.989 ± 66.431	372.946 ± 72.030	382.909 ± 69.935	380.438 ± 66.102	384.090 ± 59.353	≤0.001
CC	VAD	47.279 ± 3.293	47.958 ± 2.952	48.231 ± 2.724	47.985 ± 3.010	48.501 ± 1.584	0.101
Haller	Mean Diameter	97.161 ± 10.162	94.839 ± 8.462	93.516 ± 7.628	93.097 ± 7.709	93.083 ± 7.790	≤0.001

Values are presented as mean ± standard deviation. SCP = superficial capillary plexus; DCP = deep capillary plexus; CC = choriocapillaris; VAD = vessel area density; VLD = vessel length density. *p*-values were derived from linear mixed-effects models including random intercepts for subjects.

**Table 3:** Significant inter-layer correlations of postoperative vascular changes.

Parameter	Layer Pair	Spearman r	*p*-Value
VAD	SCP–DCP	0.504	0.01
VAD	DCP–CC	0.532	0.006
VAD	CC–Haller	−0.647	≤0.001
Mean Diameter	SCP–DCP	−0.224	0.031

SCP = superficial capillary plexus; DCP = deep capillary plexus; CC = choriocapillaris; VAD = vessel area density. Changes in vascular parameters were calculated as differences from baseline. *p*-values were calculated using two-sided Spearman correlation analysis.

**Table 4:** Mixed-effects model analysis of longitudinal OCTA parameter changes according to clinical factors.

Clinical Factor	Layer	Parameter	β (Time × Factor)	*p*-Value
Retrobulbar	CC	VAD	−0.526	0.015
Sex	CC	VAD	−0.366	0.034
Sex	CC	Nodes	−38.76	0.028
Sex	DCP	Mean Diameter	−0.224	0.031
Sex	Haller	Nodes	12.86	0.009
Sex	Haller	Total Length	2.66	0.011
Sex	Haller	VAD	0.382	0.013
Sex	Haller	VLD (%)	0.13	0.014

DCP = deep capillary plexus; CC = choriocapillaris; Haller = Haller layer; VAD = vessel area density; VLD = vessel length density.

## Data Availability

The datasets generated and/or analyzed during the current study are available from the corresponding author upon reasonable request. Data are not publicly available due to privacy and institutional restrictions.

## Supplementary Materials

The following supporting information can be downloaded at https://www.mdpi.com/article/10.3390/tomography12030042/s1. Table S1: Summary and Definitions of OCTA Metrics; Table S2: Spearman correlation analysis of inter-layer changes in OCTA parameters following phacoemulsification; Table S3: Mixed-effects model results for longitudinal OCTA parameter changes according to clinical factors; Table S4: Mixed-effects model results for longitudinal OCTA parameter changes according to clinical factors adjusted by signal strength. Figure S1: Signal strength index (SSI) distribution and its temporal relationship with retinal vascular parameters. (A) Distribution of signal strength index (SSI) values across study time points (baseline, 1 day, 1 week, 1 month, and 2 months) shown as violin plots. Baseline scans were required to have SSI ≥ 8, and overall SSI increased slightly after surgery. (B) Temporal trends of mean SSI alongside mean superficial capillary plexus (SCP) and deep capillary plexus (DCP) vessel area density (VAD). Although SSI increased after cataract surgery, the longitudinal increases in SCP and DCP VAD were not strictly proportional to SSI changes, supporting that the observed vascular trends were not solely explained by improved image quality.
