# Supplementary material for: Automated Longitudinal Quantification of Retinal and Choroidal Vascular Changes After Phacoemulsification
Source: Tomography. 2026 Mar 19;12(3):42. doi: 10.3390/tomography12030042 (PMC13029883; doi:10.3390/tomography12030042)
Supplement: Supplementary file 1 [file tomography-12-00042-s001.zip › Supplementary Table S1.pdf]

Supplementary Table S1. Summary and Definitions of OCTA Metrics

Category	Metric (Abbreviation)	Definition and Methodology
Density	Vessel Area Density (VAD)	The percentage of the binarized image area occupied by perfused blood vessels.
	Vessel Length Density (VLD)	The total length of vessels (calculated from the skeletonized centerline) per unit area.
Morphology	Mean/Median Vessel Diameter	The average thickness of vessels, estimated using a local thickness algorithm on binarized images.
	Mean/Median Vessel Length	The average length of individual identified vessel segments in the skeletonized image.
	Mean Tortuosity	A measure of vessel curvature and twisting, quantified via the OCTAVA pipeline.
Complexity	Node Numbers	The total number of connection points where multiple vessel segments meet.
	Total Vessel Length	The sum of the lengths of all detected vessel segments within the en face slab.
Distribution	Skew / Kurtosis	Statistical measures describing the asymmetry and "tailedness" of vessel diameter or length distributions.
