# Supplementary material for: Automated Longitudinal Quantification of Retinal and Choroidal Vascular Changes After Phacoemulsification
Source: Tomography. 2026 Mar 19;12(3):42. doi: 10.3390/tomography12030042 (PMC13029883; doi:10.3390/tomography12030042)
Supplement: Supplementary file 1 [file tomography-12-00042-s001.zip › Supplementary Table S2.pdf]

Supplementary Table S2. Spearman correlation analysis of inter-layer changes in OCTA parameters following phacoemulsification

Parameter	Layer A	Layer B	r, 1day	p, 1day	r, 1week	p, 1week	r, 1month	p, 1month	r, 2month	p, 2month
Mean diameter	SCP	DEEP	0.157	0.398	0.164	0.379	0.103	0.580	0.341	0.095
	SCP	CC	0.189	0.308	-0.039	0.835	-0.081	0.667	0.095	0.652
	SCP	HALLER	0.489	0.008	0.360	0.060	0.519	0.005	0.465	0.045
	DEEP	CC	0.288	0.116	0.405	0.024	0.539	0.002	0.434	0.030
	DEEP	HALLER	0.474	0.011	0.387	0.042	0.161	0.413	0.428	0.067
	CC	HALLER	0.224	0.252	0.197	0.316	0.022	0.913	0.317	0.187
Vessel area density	SCP	DEEP	0.720	0.000	0.689	0.000	0.566	0.001	0.504	0.010
	SCP	CC	-0.099	0.595	0.109	0.558	0.099	0.596	0.176	0.400
	SCP	HALLER	0.068	0.731	0.031	0.875	-0.042	0.831	0.112	0.647
	DEEP	CC	0.046	0.804	0.413	0.021	0.435	0.014	0.532	0.006
	DEEP	HALLER	0.216	0.270	-0.157	0.426	0.005	0.978	0.130	0.596
	CC	HALLER	-0.712	0.000	-0.655	0.000	-0.491	0.008	-0.525	0.021
Vessel length density (%)	SCP	DEEP	0.723	0.000	0.437	0.014	0.668	0.000	0.367	0.071
	SCP	CC	0.081	0.665	-0.235	0.203	-0.247	0.180	-0.126	0.548
	SCP	HALLER	0.482	0.009	0.138	0.485	0.138	0.482	0.323	0.178
	DEEP	CC	-0.020	0.917	0.037	0.844	-0.035	0.850	0.075	0.723
	DEEP	HALLER	0.307	0.112	-0.109	0.579	0.101	0.610	0.102	0.679
	CC	HALLER	-0.194	0.323	-0.358	0.062	0.042	0.834	-0.111	0.652
Mean tortuosity	SCP	DEEP	0.227	0.244	0.363	0.058	0.100	0.613	0.081	0.721
	SCP	CC	0.465	0.019	0.083	0.708	0.048	0.815	0.355	0.114
	SCP	HALLER	0.042	0.838	0.000	0.999	0.288	0.183	0.125	0.633
	DEEP	CC	0.050	0.815	-0.054	0.802	0.154	0.452	-0.042	0.865
	DEEP	HALLER	-0.092	0.661	0.054	0.792	0.137	0.532	0.294	0.252
	CC	HALLER	-0.046	0.831	0.322	0.134	-0.084	0.710	0.303	0.237
Nodes	SCP	DEEP	0.602	0.000	0.623	0.000	0.507	0.004	0.357	0.079
	SCP	CC	-0.308	0.092	0.004	0.985	-0.089	0.633	0.079	0.708
	SCP	HALLER	0.346	0.071	0.095	0.632	-0.082	0.680	0.035	0.887
	DEEP	CC	-0.099	0.595	0.168	0.367	0.070	0.708	0.124	0.555
	DEEP	HALLER	0.183	0.350	-0.286	0.140	0.030	0.881	0.009	0.972
	CC	HALLER	-0.604	0.001	-0.623	0.000	-0.399	0.035	-0.623	0.004
Total length	SCP	DEEP	0.721	0.000	0.439	0.013	0.665	0.000	0.367	0.071
	SCP	CC	0.081	0.663	-0.243	0.187	-0.247	0.180	-0.118	0.575
	SCP	HALLER	0.483	0.009	0.127	0.520	0.149	0.450	0.323	0.178
	DEEP	CC	-0.017	0.928	0.040	0.833	-0.029	0.878	0.091	0.666

DEEP	HALLER	0.308	0.111	-0.100	0.612	0.104	0.598	0.102	0.679
CC	HALLER	-0.195	0.320	-0.352	0.066	0.039	0.844	-0.114	0.642

SCP = superficial capillary plexus; DCP (DEEP) = deep capillary plexus; CC = choriocapillaris; Haller = Haller layer; VAD = vessel area density; VLD = vessel length density.

Spearman correlation (r) analysis was used to assess inter-layer associations due to non-normal distribution of change values.

A p-value < 0.05 was considered statistically significant.
