# Supplementary material for: Automated Longitudinal Quantification of Retinal and Choroidal Vascular Changes After Phacoemulsification
Source: Tomography. 2026 Mar 19;12(3):42. doi: 10.3390/tomography12030042 (PMC13029883; doi:10.3390/tomography12030042)
Supplement: Supplementary file 1 [file tomography-12-00042-s001.zip › Supplementary Table S3.pdf]

Supplementary Table S3. Mixed-effects model results for longitudinal OCTA parameter changes according to clinical factors

Clinical factor	Layer	Parameter	Primary			Sensitivity		
			β (Time × factor)	95% CI	p-value	β (Time × factor)	95% CI	p-value
Diabetes mellitus	SCP	Mean diameter	-0.008	[-0.021, 0.005]	0.235	-0.002	[-0.014, 0.011]	0.79
		VAD	0.009	[-0.012, 0.031]	0.402	0.001	[-0.019, 0.021]	0.943
		VLD (%)	0.011	[-0.006, 0.028]	0.202	0.004	[-0.014, 0.022]	0.643
		Mean tortuosity	0	[-0.000, 0.000]	0.868	0	[-0.000, 0.000]	0.729
		Nodes	1.278	[-1.828, 4.384]	0.42	0.137	[-3.058, 3.332]	0.933
		Total length	0.228	[-0.124, 0.579]	0.204	0.085	[-0.278, 0.447]	0.647
	DCP	Mean diameter	-0.004	[-0.015, 0.008]	0.555	-0.004	[-0.017, 0.010]	0.591
		VAD	0.017	[-0.025, 0.059]	0.419	0.013	[-0.033, 0.059]	0.583
		VLD (%)	0.003	[-0.007, 0.013]	0.596	0.001	[-0.010, 0.012]	0.867
		Mean tortuosity	0	[-0.000, 0.000]	0.791	0	[-0.000, 0.000]	0.708
		Nodes	0.686	[-4.256, 5.629]	0.785	-0.396	[-5.552, 4.760]	0.88
		Total length	0.138	[-0.369, 0.645]	0.594	0.047	[-0.501, 0.596]	0.866
	CC	Mean diameter	0.008	[-0.012, 0.029]	0.43	0.016	[-0.007, 0.039]	0.176
		VAD	-0.004	[-0.023, 0.015]	0.665	0.007	[-0.013, 0.027]	0.497
		VLD (%)	-0.013	[-0.027, 0.001]	0.061	-0.015	[-0.031, 0.001]	0.062
		Mean tortuosity	0	[-0.000, 0.000]	0.638	0	[-0.000, 0.000]	0.729
		Nodes	-1.197	[-3.401, 1.007]	0.287	-0.729	[-3.121, 1.663]	0.55
		Total length	-0.269	[-0.550, 0.012]	0.06	-0.309	[-0.633, 0.016]	0.062
	Haller	Mean diameter	0.03	[-0.110, 0.169]	0.676	0.064	[-0.097, 0.225]	0.435
		VAD	0.008	[-0.019, 0.036]	0.555	0.007	[-0.025, 0.040]	0.663
		VLD (%)	0.003	[-0.007, 0.014]	0.545	0	[-0.012, 0.012]	0.995
		Mean tortuosity	0	[-0.000, 0.000]	0.649	0	[-0.000, 0.000]	0.731
		Nodes	0.01	[-1.065, 1.084]	0.986	-0.229	[-1.476, 1.018]	0.719
		Total length	0.067	[-0.152, 0.286]	0.548	-0.001	[-0.254, 0.251]	0.993
Sex (male)	SCP	Mean diameter	-0.012	[-0.026, 0.002]	0.084	-0.005	[-0.018, 0.008]	0.457
		VAD	0.007	[-0.016, 0.031]	0.555	-0.003	[-0.024, 0.019]	0.805
		VLD (%)	0.005	[-0.014, 0.024]	0.606	-0.004	[-0.023, 0.016]	0.715
		Mean tortuosity	0	[-0.000, 0.000]	0.115	0	[-0.000, -0.000]	0.016
		Nodes	1.768	[-1.603, 5.138]	0.304	0.192	[-3.293, 3.677]	0.914
		Total length	0.101	[-0.283, 0.486]	0.605	-0.074	[-0.469, 0.322]	0.716
	DCP	Mean diameter	0.009	[-0.003, 0.022]	0.146	0.009	[-0.005, 0.023]	0.212
		VAD	0.009	[-0.036, 0.055]	0.69	-0.011	[-0.061, 0.039]	0.659
		VLD (%)	-0.001	[-0.012, 0.010]	0.907	-0.006	[-0.018, 0.006]	0.34
		Mean tortuosity	0	[-0.000, 0.000]	0.82	0	[-0.000, 0.000]	0.996
		Nodes	-0.084	[-5.450, 5.283]	0.976	-2.826	[-8.394, 2.743]	0.32
		Total length	-0.03	[-0.581, 0.521]	0.915	-0.287	[-0.880, 0.306]	0.343
	CC	Mean diameter	0.013	[-0.010, 0.035]	0.267	0.009	[-0.016, 0.035]	0.462
		VAD	0.017	[-0.003, 0.037]	0.093	0.023	[0.002, 0.044]	0.029
		VLD (%)	0.003	[-0.012, 0.018]	0.712	0.005	[-0.013, 0.022]	0.609
		Mean tortuosity	0	[-0.000, 0.000]	0.497	0	[-0.000, 0.000]	0.143

		Nodes	1.444	[-0.943, 3.832]	0.236	2.407	[-0.140, 4.954]	0.064
		Total length	0.057	[-0.252, 0.366]	0.717	0.093	[-0.265, 0.450]	0.612
	Haller	Mean diameter	0.178	[0.022, 0.334]	0.025	0.191	[0.012, 0.370]	0.037
		VAD	-0.02	[-0.052, 0.011]	0.201	-0.026	[-0.062, 0.011]	0.173
		VLD (%)	-0.012	[-0.024, -0.000]	0.046	-0.015	[-0.029, -0.002]	0.028
		Mean tortuosity	0	[-0.000, 0.000]	0.907	0	[-0.000, 0.000]	0.993
		Nodes	-1.904	[-3.080, -0.727]	0.002	-2.229	[-3.561, -0.897]	0.001
		Total length	-0.25	[-0.497, -0.003]	0.047	-0.312	[-0.592, -0.033]	0.028
Retrobulbar Anesthesia	SCP	Mean diameter	-0.005	[-0.024, 0.015]	0.647	-0.004	[-0.023, 0.014]	0.652
		VAD	0.004	[-0.028, 0.037]	0.806	0	[-0.030, 0.030]	0.998
		VLD (%)	0.012	[-0.014, 0.038]	0.384	0.012	[-0.015, 0.038]	0.395
		Mean tortuosity	0	[-0.000, 0.000]	0.606	0	[-0.000, 0.000]	0.964
		Nodes	1.149	[-3.503, 5.802]	0.628	0.702	[-4.060, 5.463]	0.773
		Total length	0.237	[-0.295, 0.769]	0.383	0.237	[-0.308, 0.781]	0.394
	DCP	Mean diameter	-0.002	[-0.019, 0.016]	0.852	-0.002	[-0.021, 0.018]	0.857
		VAD	-0.016	[-0.078, 0.046]	0.62	-0.019	[-0.087, 0.049]	0.582
		VLD (%)	-0.005	[-0.020, 0.010]	0.482	-0.006	[-0.022, 0.010]	0.461
		Mean tortuosity	0	[-0.000, 0.000]	0.729	0	[-0.000, 0.000]	0.52
		Nodes	-1.532	[-8.883, 5.820]	0.683	-2.59	[-10.263, 5.084]	0.508
		Total length	-0.27	[-1.023, 0.484]	0.483	-0.306	[-1.119, 0.507]	0.461
	CC	Mean diameter	-0.008	[-0.039, 0.023]	0.615	-0.014	[-0.049, 0.021]	0.424
		VAD	-0.022	[-0.050, 0.006]	0.123	-0.021	[-0.051, 0.008]	0.156
		VLD (%)	0.003	[-0.018, 0.024]	0.812	0.01	[-0.015, 0.034]	0.44
		Mean tortuosity	0	[0.000, 0.000]	0.01	0	[0.000, 0.000]	0.002
		Nodes	-1.884	[-5.222, 1.453]	0.269	-1.107	[-4.732, 2.517]	0.549
		Total length	0.051	[-0.378, 0.481]	0.815	0.195	[-0.302, 0.691]	0.442
	Haller	Mean diameter	-0.074	[-0.340, 0.192]	0.587	-0.069	[-0.378, 0.239]	0.661
		VAD	0.027	[-0.026, 0.081]	0.31	0.045	[-0.018, 0.109]	0.16
		VLD (%)	0.009	[-0.011, 0.030]	0.38	0.011	[-0.013, 0.034]	0.38
		Mean tortuosity	0	[-0.000, 0.001]	0.423	0	[-0.000, 0.001]	0.323
		Nodes	0.862	[-1.205, 2.930]	0.414	1.246	[-1.148, 3.640]	0.308
		Total length	0.188	[-0.232, 0.607]	0.38	0.216	[-0.267, 0.700]	0.38

SCP, superficial capillary plexus; DCP, deep capillary plexus; CC, choriocapillaris; VAD, vessel area density; VLD, vessel length density

β represents the estimated difference in time slope (time × factor interaction term) from a linear mixed-effects model with random intercepts for eyes. Two-sided p-values are shown.
