# Supplementary material for: Automated Longitudinal Quantification of Retinal and Choroidal Vascular Changes After Phacoemulsification
Source: Tomography. 2026 Mar 19;12(3):42. doi: 10.3390/tomography12030042 (PMC13029883; doi:10.3390/tomography12030042)
Supplement: Supplementary file 1 [file tomography-12-00042-s001.zip › Supplementary Table S4.pdf]

Supplementary Table S4. Mixed-effects model results for longitudinal OCTA parameter changes according to clinical factors adjusted by signal strength

Clinical factor	Layer	Parameter	Primary			Sensitivity		
			β (Time × factor)	95% CI	p-value	β (Time × factor)	95% CI	p-value
Diabetes mellitus	SCP	Mean diameter	-0.008	[-0.021, 0.005]	0.233	-0.002	[-0.014, 0.011]	0.781
		VAD	0.008	[-0.013, 0.030]	0.451	-0.001	[-0.020, 0.019]	0.958
		VLD (%)	0.011	[-0.006, 0.028]	0.221	0.004	[-0.014, 0.021]	0.697
		Mean tortuosity	0	[-0.000, 0.000]	0.949	0	[-0.000, 0.000]	0.916
		Nodes	1.15	[-1.930, 4.230]	0.464	-0.037	[-3.222, 3.148]	0.982
		Total length	0.217	[-0.132, 0.567]	0.223	0.071	[-0.291, 0.433]	0.701
	DCP	Mean diameter	-0.004	[-0.015, 0.008]	0.525	-0.004	[-0.017, 0.009]	0.533
		VAD	0.015	[-0.026, 0.056]	0.467	0.009	[-0.035, 0.054]	0.678
		VLD (%)	0.002	[-0.008, 0.012]	0.652	0	[-0.011, 0.011]	0.966
		Mean tortuosity	0	[-0.000, 0.000]	0.798	0	[-0.000, 0.000]	0.682
		Nodes	0.499	[-4.394, 5.392]	0.841	-0.713	[-5.793, 4.367]	0.783
		Total length	0.115	[-0.384, 0.614]	0.651	0.012	[-0.529, 0.553]	0.964
	CC	Mean diameter	0.007	[-0.013, 0.028]	0.47	0.014	[-0.008, 0.037]	0.207
		VAD	-0.005	[-0.023, 0.013]	0.58	0.005	[-0.014, 0.024]	0.59
		VLD (%)	-0.013	[-0.027, 0.001]	0.064	-0.015	[-0.031, 0.001]	0.068
		Mean tortuosity	0	[-0.000, 0.000]	0.611	0	[-0.000, 0.000]	0.777
		Nodes	-1.231	[-3.441, 0.980]	0.275	-0.793	[-3.196, 1.611]	0.518
		Total length	-0.267	[-0.548, 0.015]	0.064	-0.303	[-0.628, 0.022]	0.068
	Haller	Mean diameter	0.027	[-0.112, 0.166]	0.705	0.059	[-0.102, 0.221]	0.469
		VAD	0.009	[-0.018, 0.036]	0.52	0.008	[-0.025, 0.041]	0.631
		VLD (%)	0.004	[-0.007, 0.014]	0.494	0.001	[-0.012, 0.013]	0.91
		Mean tortuosity	0	[-0.000, 0.000]	0.632	0	[-0.000, 0.000]	0.726
		Nodes	0.055	[-1.008, 1.119]	0.919	-0.156	[-1.395, 1.082]	0.804
		Total length	0.076	[-0.143, 0.294]	0.497	0.014	[-0.238, 0.266]	0.912
Sex (male)	SCP	Mean diameter	-0.012	[-0.026, 0.002]	0.087	-0.005	[-0.018, 0.008]	0.461
		VAD	0.008	[-0.015, 0.031]	0.504	-0.003	[-0.025, 0.018]	0.767
		VLD (%)	0.006	[-0.013, 0.024]	0.554	-0.003	[-0.023, 0.016]	0.722
		Mean tortuosity	0	[-0.000, 0.000]	0.059	0	[-0.000, -0.000]	0.003
		Nodes	1.892	[-1.448, 5.231]	0.267	0.17	[-3.300, 3.640]	0.924
		Total length	0.116	[-0.267, 0.498]	0.553	-0.071	[-0.465, 0.323]	0.723
	DCP	Mean diameter	0.01	[-0.003, 0.022]	0.134	0.009	[-0.005, 0.023]	0.215
		VAD	0.012	[-0.033, 0.056]	0.612	-0.012	[-0.060, 0.037]	0.635
		VLD (%)	0	[-0.011, 0.011]	0.973	-0.006	[-0.018, 0.006]	0.322
		Mean tortuosity	0	[-0.000, 0.000]	0.809	0	[-0.000, 0.000]	0.999
		Nodes	0.112	[-5.200, 5.424]	0.967	-2.884	[-8.361, 2.593]	0.302
		Total length	-0.006	[-0.549, 0.536]	0.982	-0.292	[-0.876, 0.291]	0.326
	CC	Mean diameter	0.014	[-0.008, 0.036]	0.22	0.009	[-0.015, 0.034]	0.451

		VAD	0.018	[-0.001, 0.038]	0.065	0.023	[0.003, 0.043]	0.025
		VLD (%)	0.003	[-0.012, 0.018]	0.728	0.005	[-0.013, 0.022]	0.604
		Mean tortuosity	0	[-0.000, 0.000]	0.488	0	[-0.000, 0.000]	0.155
		Nodes	1.478	[-0.916, 3.873]	0.226	2.395	[-0.160, 4.951]	0.066
		Total length	0.054	[-0.256, 0.363]	0.733	0.094	[-0.263, 0.451]	0.607
	Haller	Mean diameter	0.179	[0.022, 0.335]	0.025	0.188	[0.008, 0.368]	0.04
		VAD	-0.02	[-0.051, 0.011]	0.204	-0.025	[-0.062, 0.012]	0.194
		VLD (%)	-0.012	[-0.024, -0.000]	0.046	-0.015	[-0.028, -0.001]	0.034
		Mean tortuosity	0	[-0.000, 0.000]	0.914	0	[-0.000, 0.000]	0.991
		Nodes	-1.899	[-3.062, -0.737]	0.001	-2.162	[-3.487, -0.838]	0.001
		Total length	-0.249	[-0.496, -0.003]	0.047	-0.301	[-0.580, -0.022]	0.035
Retrobulbar Anesthesia	SCP	Mean diameter	-0.004	[-0.024, 0.016]	0.669	-0.004	[-0.023, 0.015]	0.671
		VAD	0.008	[-0.024, 0.040]	0.634	0.003	[-0.027, 0.033]	0.835
		VLD (%)	0.014	[-0.012, 0.040]	0.301	0.013	[-0.013, 0.040]	0.324
		Mean tortuosity	0	[-0.000, 0.000]	0.816	0	[-0.000, 0.000]	0.616
		Nodes	1.631	[-2.978, 6.240]	0.488	1.132	[-3.606, 5.870]	0.64
		Total length	0.28	[-0.250, 0.809]	0.301	0.274	[-0.269, 0.817]	0.323
	DCP	Mean diameter	0	[-0.018, 0.017]	0.961	0	[-0.020, 0.020]	0.994
		VAD	-0.006	[-0.067, 0.055]	0.84	-0.008	[-0.075, 0.058]	0.802
		VLD (%)	-0.003	[-0.018, 0.011]	0.654	-0.004	[-0.020, 0.012]	0.63
		Mean tortuosity	0	[-0.000, 0.000]	0.759	0	[-0.000, 0.000]	0.582
		Nodes	-0.67	[-7.962, 6.622]	0.857	-1.578	[-9.153, 5.998]	0.683
		Total length	-0.17	[-0.913, 0.573]	0.655	-0.197	[-1.000, 0.605]	0.63
	CC	Mean diameter	-0.005	[-0.035, 0.026]	0.77	-0.01	[-0.045, 0.024]	0.556
		VAD	-0.018	[-0.045, 0.009]	0.2	-0.017	[-0.046, 0.012]	0.256
		VLD (%)	0.002	[-0.019, 0.023]	0.85	0.009	[-0.016, 0.033]	0.481
		Mean tortuosity	0	[0.000, 0.000]	0.012	0	[0.000, 0.000]	0.002
		Nodes	-1.76	[-5.124, 1.604]	0.305	-0.934	[-4.594, 2.726]	0.617
		Total length	0.041	[-0.392, 0.473]	0.853	0.179	[-0.321, 0.679]	0.483
	Haller	Mean diameter	-0.067	[-0.333, 0.199]	0.62	-0.064	[-0.373, 0.244]	0.684
		VAD	0.026	[-0.027, 0.079]	0.34	0.045	[-0.019, 0.108]	0.169
		VLD (%)	0.008	[-0.012, 0.029]	0.418	0.01	[-0.013, 0.034]	0.399
		Mean tortuosity	0	[-0.000, 0.001]	0.418	0	[-0.000, 0.001]	0.326
		Nodes	0.768	[-1.276, 2.812]	0.462	1.183	[-1.189, 3.556]	0.328
		Total length	0.172	[-0.245, 0.590]	0.419	0.207	[-0.274, 0.688]	0.399

SCP, superficial capillary plexus; DCP, deep capillary plexus; CC, choriocapillaris; VAD, vessel area density; VLD, vessel length density

β represents the estimated difference in time slope (time × factor interaction term) from a linear mixed-effects model with random intercepts for eyes. Two-sided p-values are shown.
