# Supplementary figures and images for: Automated Longitudinal Quantification of Retinal and Choroidal Vascular Changes After Phacoemulsification
Source: Tomography. 2026 Mar 19;12(3):42. doi: 10.3390/tomography12030042 (PMC13029883; doi:10.3390/tomography12030042)

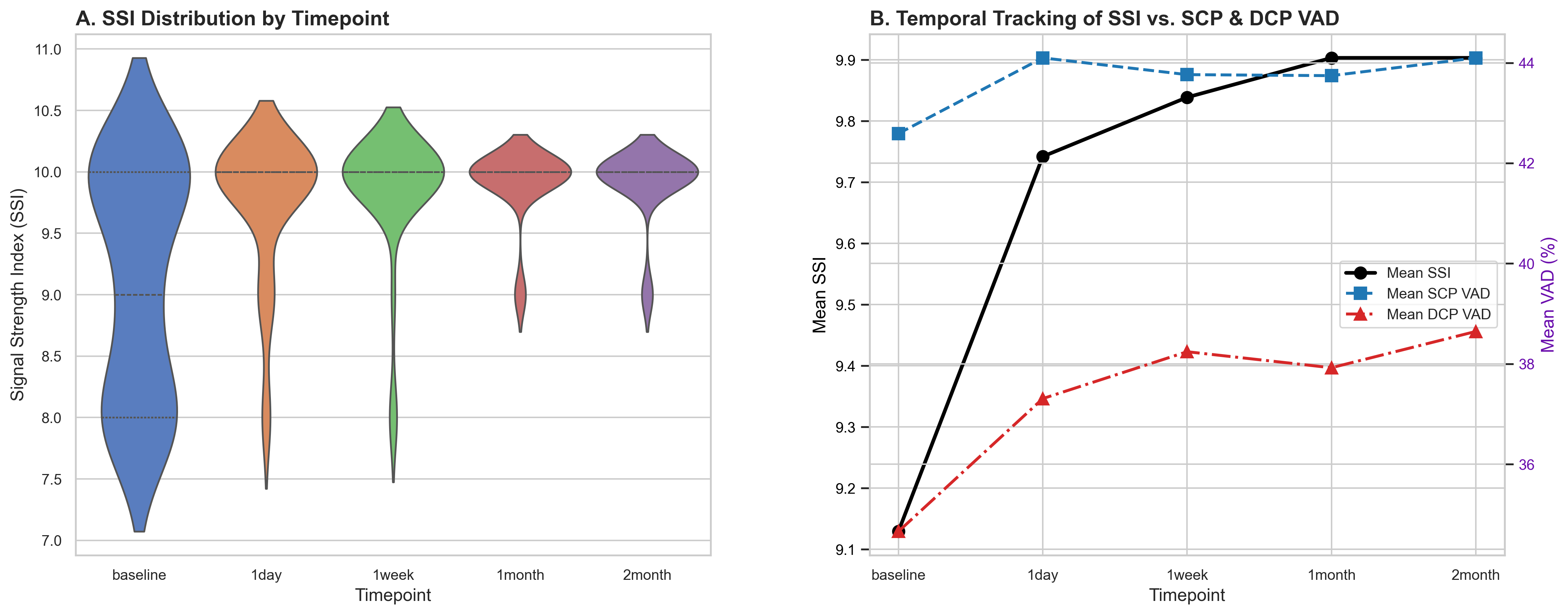

Supplement: Supplementary file 1 [file tomography-12-00042-s001.zip › Supplementary_Figure_S1_Combined.png]
